# Genetically modified α-amylase inhibitor peas are not specifically allergenic in mice

**DOI:** 10.1186/2045-7022-3-S3-P84

**Published:** 2013-07-25

**Authors:** D Reiner, R Lee, TJ Higgins, MM Epstein

**Affiliations:** 1 Department of Dermatology, Division of Immunology, Allergy and Infectious Diseases, Medical University of Vienna, Vienna, Austria; 2 Plant Industry, CSIRO, Canberra, Australia

## Background

Weevils can devastate food legumes in developing countries, but genetically modified peas (*Pisum sativum*), chickpeas and cowpeas expressing the gene for alpha-amylase inhibitor-1 (αAI) from the common bean (*Phaseolus vulgaris*) are completely protected from weevil destruction. αAI is seed-specific, accumulated at high levels and undergoes post-translational modification as it traverses the seed endomembrane system. This modification was thought to be responsible for the reported allergenicity in mice of the transgenic pea but not the bean.

## Methods

To evaluate whether consumption of bean and αAI pea seed meals generated allergic responses to αAI, we fed mice αAI transgenic peas, non-transgenic (nGM) peas, Tendergreen bean (sourse of αAI gene) and Pinto bean. MIce received raw or heat-treated seed meal diluted in PBS twice weekly for 4 consecutive weeks, followed by 50 µg of αAI i.n. We then measured allergic airway and lung inflammation, mucus hypersecretion and antibody production.

## Results

In this study we observed that both the transgenic legumes and non-transgenic beans were allergenic in BALB/c mice. Even consuming non-transgenic peas lacking αAI led to an anti-αAI response due to a cross-reactive response to pea lectin. Our data demonstrate that αAI transgenic peas are not more allergenic than beans or non-transgenic peas in mice.

## Conclusion

This study illustrates the importance of repeat experiments in independent laboratories and the potential for unexpected crossreactive allergic responses upon consumption of plant products in mice.

## Disclosure of interest

None declared.

